# Nurses’ challenges and strategies for safeguarding care quality and safety: A qualitative study on situated resilience

**DOI:** 10.1016/j.ijnsa.2025.100365

**Published:** 2025-06-11

**Authors:** Mariëlle Van Mersbergen-de Bruin, Catharina Van Oostveen, Anne Marie Weggelaar-Jansen

**Affiliations:** a Tranzo, Tilburg School of Social and Behavioural Sciences, Tilburg University, Tilburg, the Netherlands; b Erasmus School of Health Policy and Management, Department Health Services Management & Organisation, Erasmus University Rotterdam, Rotterdam, the Netherlands

**Keywords:** Patient safety, Healthcare quality, Nursing, Nursing research, Healthcare delivery, Qualitative research

## Abstract

**Background:**

Most healthcare delivery succeeds in safeguarding high-quality care and safety. This is largely due to the adaptive capacity and situated resilience work of healthcare professionals as nurses who keeps things on track*.* However, much of their situated resilience work in complex everyday practice remains hidden or is done behind the scenes.

**Objective:**

To explore situated resilience in everyday nursing practice and shed light on the often invisible efforts of nurses as they manage immediate challenges and navigate complex processes to ensure care quality and safety.

**Design:**

A qualitative design.

**Setting(s):**

The surgical and an ambulatory care team of an urban, 600-bed Dutch teaching hospital.

**Participants:**

Nurses (*N* = 37), nurse practitioners (*N* = 2), managers (*N* = 5),

**Methods:**

Data were collected through 80 hours of non-participant non-participant observations, three semi-structured interviews with nurses and one monodisciplinary (i.e. nurses) focus group. Thereafter, two multidisciplinary focus groups were conducted. Data were analysed using thematic analysis. The research protocol was approved by the ethical review board of Erasmus University Rotterdam under number ETH2122-0079.

**Results:**

Nurses are dedicated providing the high-quality care that ensures patient safety. In daily practice they face challenges that require them to make changes to system standards. We identified three key triggers for change and emerging strategies to handle these triggers: 1) when standard risk assessment does not fit nursing practice, nurses a) seem to comply with the system, b) adopt an investigative, attentive approach. 2) when protocols and guidelines do not align with daily practices, nurses a) proactively identify potential and actual changes, b) find the "golden mean" through relational negotiation and patient advocacy. 3) when nurses and other healthcare professionals hold differing values on care quality and safety, nurses a) find allies, and b) applied various indirect means.

**Conclusions:**

Our study reveals that situated resilience in nursing is not only a television series of actions to fix misalignments or to deal with unexpected change. It unfolds as a relational process in which nurses adapt their behaviour intuitively according to a patient’s unique situation, values, and interests. By balancing the individual patient’s needs and values with organizational systemic demands nurses exhibit situated resilience. By recognizing and supporting situated resilience practices, organizations not only enhance the quality of daily practice but also structurally strengthen their adaptive and resilient capacities. Further research into the role of nurses in system-level resilience and the impact of experience on resilience behaviour is needed.

**Social media abstract:**

Nurses exhibit resilience by balancing patient’s needs with organizational systemic demands, and aligning differing values in a relational process @ijnsa2025

**What is already known**

• Nurses ensure safe care by adapting practices, showing situated resilience.

• Nurses’ resilience often works behind the scenes as the "glue in the system."

• Situated resilience involves actions to prevent or address misalignments.

What this paper adds

• *Situated resilience is relational, driven by patients’ unique needs and values.*

• *Nurses balance care needs with system demands, requiring compassion and empathy.*

Alt-text: Unlabelled box

## 1 Background

Traditional approaches to patient safety focus on error-based improvements and learning from past adverse events (Hollnagel, 2014; Peerally et al., 2017). This reactive strategy, based on linear cause-effect analysis is prone to hindsight bias, and often results in blaming the professional(s) or organisational structures (Hugh and Dekker, 2009). Despite many undesired outcomes, most healthcare delivery succeeds in safeguarding quality care and safety (Braithwaite et al., 2015; Hollnagel, 2014). This success is largely due to the adaptive capacity of healthcare professionals, such as nurses (Braithwaite et al., 2015; Hollnagel, 2014). In this paper, we explore how nurses constantly adapt their practices to meet patient needs and ensure safe, high-quality care, even when organisational structures, processes, policies, and resources fail to support their daily work. This exploration is rooted in a patient-safety approach known as "Resilience in Healthcare" also referred to as "Safety II" that emphasises the importance of flexible adaptations to meet the complex challenges of current healthcare environments (Hollnagel, 2014; Lyng et al., 2022; Wiig and Fahlbruch, 2019).

Resilience in Healthcare employs a systemic view of patient safety that is grounded in human-factors engineering, psychology, environmental science, and complexity science (Hollnagel, 2014; Wiig and Fahlbruch, 2019). Resilience in Healthcare highlights the individual’s capacity to adapt, restore, or transform practices while maintaining quality care amid persistent challenges and continuous changes in healthcare systems (Behrens et al., 2022; Wiig and Fahlbruch, 2019). The approach involves non-linear interactions among both actors and institutions processes, rules and regulations, including professionals, teams, organisations, and society at large, as well as (national) regulatory bodies, funding organisations, professional bodies etc. and their (infra)structures (Hollnagel, 2014; Wiig and Fahlbruch, 2019). The interactions between actors and factors create unexpected and unforeseen situations, leading to desired and undesired outcomes (Braithwaite et al., 2015; Hollnagel, 2014; Mannion and Braithwaite, 2017). To effectively handle these situations demands adaptations of practices, processes, and standards across all levels of the healthcare sector (Weick, Karl E. and Sutcliffe, 2017; Wiig and Fahlbruch, 2019). Such adaptations require the resilience of all involved, an understudied topic in healthcare.

Resilience in Healthcare distinguishes three levels of interaction where resilience occurs (Macrae, 2019; Wiig and Fahlbruch, 2019). 1) The macro level or "system resilience" involves the adaptability of the entire healthcare system (Macrae, 2019; Wiig and Fahlbruch, 2019). 2) The *meso* level or "structural resilience" focuses on an organisation’s ability to adjust its infrastructures, processes, and resources to support the delivery of care quality and safety (Macrae, 2019; Wiig and Fahlbruch, 2019). 3) The micro level or "situated resilience" refers to the immediate, context-specific adaptations to daily practices healthcare professionals -such as nurses- make (Macrae, 2019; Wiig and Fahlbruch, 2019). This third level becomes critical when societal or organisational structures (e.g. procedures, rules and regulations) do not align with the professionals’ real-world practices (Allen, 2015; Hollnagel, 2014).

Despite the growing literature on various levels, empirical studies seldom report on the situated resilience of nurses dealing with the challenges of the micro level (Wears et al., 2015; Wears and Suthcliffe, 2020). Working in dynamic, complex care environments, nurses demonstrate situated resilience by proactively adapting their actions – often in collaboration with others – to manage quality and safety concerns (Göransson et al., 2024). To meet the changing demands and constraints of these settings, nurses consciously make trade-offs between efficiency and thoroughness, which Hollnagel (2009) has coined the Efficiency-Thoroughness Trade-Off versus the Thoroughness- Efficiency Trade-Off (ETTO-TETO balance). Balancing efficiency and thoroughness and vice versa by deviating from the established system (Hollnagel, 2009) requires nurses to continuously monitor their practices to effectively manage the risks while ensuring high-quality, safe patient care (Allen, 2015). Through collaborative reflection, nurses learn how to improve current actions and inform future decisions (Hollnagel, 2014; Raelin, 2016). Clearly, nurses play a crucial yet often invisible role in the adaptive capacity of healthcare systems. Despite nurses being described in the literature as "the glue in the system that keeps things on track" (Allen, 2015), much of their situated resilience work remains hidden in everyday practice, given that it often is done behind the scenes (Allen, 2015; de Kok et al., 2023; Tresfon et al., 2023).

Some studies have examined situated resilience in specific, complex nursing processes and shed light on the hidden adaptations nurses make in their work, for example when using physical restraints (Tresfon et al., 2023), in double-checking when administering high-risk medication (Kaya et al., 2019; Schutijser et al., 2019; van Stralen et al., 2024), when inserting intravenous infusions (Vos et al., 2020), as well as in follow-up procedures for intensive care patients after discharge (op ’t Hoog et al., 2024). This literature describes how nurses manage disruption, address risks, and justify adjustments to ways that are far more intricate and nuanced than the protocols suggest (Tresfon et al., 2023; van Stralen et al., 2024; Vos et al., 2020). For instance, Vos et al. (2020) demonstrate that nurses contribute significantly to system resilience through their immediate response to risk assessments and justify deviating from set protocols and standards because their "fixes" prevent unsafe practices. However, these adaptations are mostly done behind the scenes, keeping them invisible, not only to nurses themselves but also to the broader healthcare system (Tresfon et al., 2023). As a result, critical knowledge often fails to inform policy and protocol development, leading to a significant gap between formal protocols and real-world nursing practice (Tresfon et al., 2023). Although these studies provide useful insights into situated resilience in nursing practice, they are limited as they focus exclusively on resilience situated in a single process and the nursing practice involves many of these processes.

Everyday nursing practice is situated in an interconnected web of interacting processes and network collaborations in the current complex healthcare environments (Allen, 2015; Benner et al., 2009; Chaffee and McNeill, 2007). These circumstances make it crucial to develop understanding of situated resilience across processes, while it is especially relevant to know how nurses overcome constraints and adapt to ever-changing demands (Chaffee and McNeill, 2007). It involves analysing how nurses and colleagues – physicians, managers and other healthcare professionals – navigate and manage the challenges of interacting processes (Allen, 2015; Chaffee and McNeill, 2007). It also helps to explore the strategies nurses use to uphold their adaptive capacity (Costa et al., 2018).

To bridge this research gap, our study delved deeper into nurses’ situated resilience, aiming to make their often invisible work manifest by revealing how they manage and resolve immediate, specific challenges while navigating the complexities of everyday nursing protocols. Hence, our research question is:

*“What challenges do nurses encounter in their daily practice, and what situated resilience strategies do they employ to manage these challenges?*”

## 2 Methods

### 2.1 Design

We conducted a qualitative study that included shadowing nurses and other healthcare professionals in daily practice and held mono- and multidisciplinary (group) interviews (Czarniawska, 2007; McDonald, 2005). The study aimed to gain an understanding of how situated resilience and adaptive behaviour emerge in irregular nursing practices (Clay-Williams et al., 2020; Holloway and Wheeler, 2002a).

### 2.2 Setting

The study was conducted in a Dutch urban teaching hospital (1500 nurses; 600 beds; annually treating 200,000 clinical patients and 534,000 outpatients). This hospital was chosen as our sample for its active participation in Dutch Resilience in Healthcare and its convenient accessibility (part of the researchers’ network). Two nursing settings were purposively selected, a surgical (Ward I) and an ambulatory (Ward II) based on their high-performance scores on nurse-sensitive quality indicators regarding pain, malnutrition, delirium management, falls, and pressure ulcers (Gormley et al., 2024). Studying the high-performing teams on these two wards allowed us to identify their best practices.

### 2.3 Sample selection and participants

We used purposeful sampling to select shifts and participants likely to provide deeper insights into situated resilience in nursing practice (Holloway and Wheeler, 2002b). We started with non-participant observations ("observations") to understand how resilience unfolds in daily nursing practice (Czarniawska, 2007; McDonald, 2005). To achieve maximum variation, we selected five full (8-hour) shifts per ward, based on criteria day vs. evening shifts, presence of management, and ward busyness (Czarniawska, 2007; McDonald, 2005). When full shifts were not feasible, we combined two partial (4-hour) shifts to ensure comparable coverage and a minimum of 40 observation hours per ward — sufficient for contextual insight and identifying recurring patterns (Malterud et al., 2016; Morse, 2015). In total, we observed three full and four partial shifts at Ward I, and five full shifts at Ward II, comprising eight day shifts and six evening shifts. Ward I included five day (three full, two partial) and four evening shifts (two full, two partial), while Ward II included three day and two evening full shifts. These observations deepened our understanding of the work context and recurring responses to everyday challenges (Czarniawska, 2007; McDonald, 2005). See Table 1 for participant characteristics. We achieved maximum variation in age, experience, and educational level among respondents.

**Table 1:** Respondent characteristics for data collection. NB: No doctors/physicians participated.

		**Non-participant observations**	**Individual interview**	**Monodisciplinary group**	**Multidisciplinary group**
**N**		16	3	8	16
**Gender**	female	14	3	6	15
	male	2	–	2	1
**Age**	<21	1	–	–	1
	21–30	5	–	6	8
	31–40	1	–	2	1
	41–50	3	1	–	3
	51–60	5	2	–	2
	>60	1	–	–	1
**Experience**	<1	1	–	–	1
**in years**	1–10	4	–	6	8
	11–20	1		2	4
	>20	9	3	–	3
**Profession**	Nurse	13	3	8	11
	Head nurse	1	n/a	n/a	3
	Manager	n/a	n/a	n/a	1
	Nurse practitioner	1	n/a	n/a	1

Eligible participants for the monodisciplinary focus group and interviews were registered nurses (RNs) directly involved in patient care, with variation in experience, age, and education. Head nurses were excluded due to their more distant roles to everyday practice. The multidisciplinary focus groups included nurses, nurse practitioners, physicians, and management staff involved in patient care, aiming for broad professional diversity in backgrounds, experience, and education (Barbour, 2018; Holloway and Wheeler, 2002c), with at least one representative per discipline.

For both focus group types, we aimed for four to ten participants to foster dynamic yet focused discussions (Barbour, 2018; Holloway and Wheeler, 2002c). In Ward II, 35 nurses were invited to the monodisciplinary focus group, and eight (22.9 %) participated. In Ward I, invitations for the multidisciplinary focus group went to one physician, one nurse practitioner, one head nurse, one manager, and 35 nurses. Eight nurses, the nurse practitioner, and team leader (25.6 %) participated. In Ward II, two head nurses, one manager, one nurse practitioner, one physician, and 30 nurses were invited; three nurses, one head nurse, one manager, and the nurse practitioner (17.1 %) participated. These three nurses also joined the monodisciplinary group. Maximum variation in age, experience, and education was achieved across both focus group types, though professional variation was limited in the multidisciplinary group due to the absence of physicians.

In Ward I, low nurse availability made a monodisciplinary focus group unfeasible; instead, we conducted individual interviews using the same eligibility criteria as for the monodisciplinary focus group. We aimed pragmatically for five participants—approximately the average focus group size (Barbour, 2018)— in line with ward operations. Three of five nurses (60 %) agreed to participate.

### 2.4 Procedure

The first author emailed the ward management to explain the study aims and invite participation, whereupon the managers informed their staff and obtained consent from all involved; no staff declined to participate in the study. Eligible respondents for mono- and multidisciplinary focus groups were informed and invited via email and/or newsletter about the study objectives and focus group aims. Potential respondents could contact an independent knowledgeable individual to answer questions and could decline the invitation without giving a reason. Upon consent, we scheduled a convenient date, time and location to maximise participation.

Before the interviews, mono- and multidisciplinary focus groups, respondents were reminded of their voluntary involvement and assured of data confidentiality and their right to withdraw at any time. Their repeated oral consent was recorded.

Monodisciplinary focus group in Ward I took place conveniently in a regular team meeting. For both multidisciplinary focus groups, a dedicated time was scheduled in a quiet setting to ensure focus and minimise distractions (Holloway and Wheeler, 2002c, 2002d).

### 2.5 Data collection

**Fig. 1:**
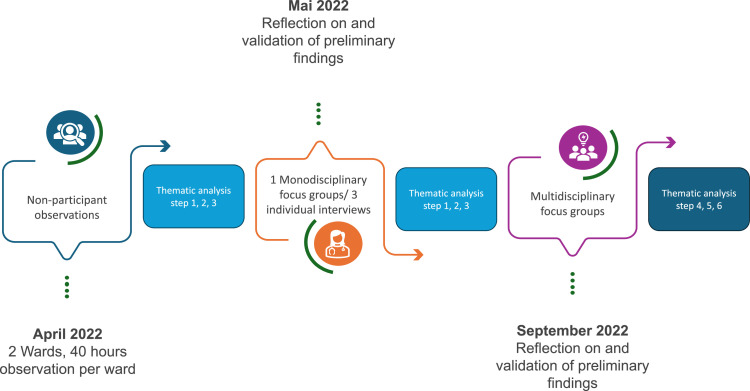
Visualization of the research process.

Data were collected from February to September 2022, starting with 80 hours of observations in total (see Fig. 1) (Czarniawska, 2007; Holloway and Wheeler, 2002e). The observations were conducted by the first author, using an observation guide specifically designed for nursing care related to pain and pain management (see Supplementary File 1). During the observations we paid attention to the broader context, including the environment and its’ atmosphere, communication, collaboration, nurses' actions and leadership practices, use of resources and technology, and relevant artefacts (DeWalt and DeWalt, 2011) — all in relation to situated resilience. Observations continued until no new insights emerged, indicating that saturation had been reached (Creswell, 2007). While observing, the first author informally discussed observations with nurses for clarification and to avoid ‘going native’ (DeWalt and DeWalt, 2011). Notes were taken by the first author during the conversations, logged events and later expanded the notes into thick descriptions to provide a detailed context for comprehensive data analysis (Denham and Onwuegbuzie, 2013).

Next, we held the semi-structured interviews (Ward I), the semi-structured monodisciplinary focus group (Ward II), and multidisciplinary focus groups on both wards. All interviews and focus groups lasted about an hour (Holloway and Wheeler, 2002c). All focus groups and interviews followed a predefined topic list (Supplementary file 2), which was enriched with insights from earlier data analysis. Observations informed the interviews in Ward I and monodisciplinary focus group in Ward II, whose analysis shaped the multidisciplinary focus groups.

Data collection and analysis occurred concurrently, allowing for data triangulation to validate and refine our analysis (Holloway and Wheeler, 2002f; Morse, 2015). These member checks and validation did not yield any new insights, indicating that data saturation had been reached (Creswell, 2007; Holloway and Wheeler, 2002f; Morse, 2015). Demographic data were collected at the start of each interview and focus group. Sessions were audio-recorded and transcribed verbatim for detailed analysis (Holloway and Wheeler, 2002f, 2002c).

### 2.6 Data analysis

Our thematic analysis was based on Braun and Clarke’s (2006) six steps (see Fig. 1). This allowed us to gain a comprehensive understanding of complex data on how nurses navigate challenges in their practices, revealing significant patterns in their adaptability (Braun and Clarke, 2006; Timmermans and Tavory, 2022).

Step1: The three authors immersed themselves independently in the data, reading the thick descriptions of the observations and the focus group and interview transcripts multiple times to understand the content and context (Braun and Clarke, 2022). We took detailed notes on emerging patterns, which facilitated preliminary insights into situated resilience in everyday nursing practice (Braun and Clarke, 2022).
Step2: Independently, we conducted the initial coding to capture diverse perspective, systematically identifying significant data segments using Atlas.ti 22.0.2 (Braun and Clarke, 2022). The coding process focussed on both explicit content and underlying themes regarding resilience (Braun and Clarke, 2022). Next, we discussed the initial list up to consensus.
Step3: We arranged the initial codes into broader themes, linking data segments to emphasise how situated resilience manifests in nurses' responses. Existing theoretical frameworks, including Resilience in Healthcare (Lyng et al., 2022; Wiig and Fahlbruch, 2019) and psychological safety (Edmonson, 2022), informed our interpretations, fostering iterative reassessment of assumptions and refinement of themes (Timmermans and Tavory, 2022).
Step4: We reviewed and refined the themes, ensuring alignment with the raw data in critical discussions (Braun and Clarke, 2006; Timmermans and Tavory, 2022). This collaborative process guaranteed that the themes were robust and reflected empirical reality.
Step5: We clearly named each defined theme to show how situated resilience operates in everyday nursing practice (Braun and Clarke, 2006).

Finally, in Step 6, we merged the themes in a cohesive narrative that connected empirical findings to theoretical insights, producing a comprehensive explanation of situated resilience in nursing and illuminating how nurses navigate complex healthcare settings (Braun and Clarke, 2006; Timmermans and Tavory, 2022).

### 2.7 Rigour and trustworthiness

To ensure rigour, we adopted a critical, reflective stance and applied systematic, transparent strategies in line with the interpretative nature of qualitative research (Holloway and Wheeler, 2002a). We used data triangulation to explore situated resilience from multiple perspectives and identify recurring patterns (Holloway and Wheeler, 2002f; Morse, 2015). Furthermore, discussing findings in interviews and focus groups supported member checking and credibility (Morse, 2015).

The thematic analysis offered a transparent and iterative framework for interpretation, informed by theoretical reflection (Braun and Clarke, 2006). Analytical triangulation was strengthened by involving two independent researchers (CO, AMW). Analytical steps one and two were done separately to reduce influence, followed by thorough discussions to mitigate bias and reach consensus, enhancing credibility and confirmability (Morse, 2015).

All authors wee former nurses. MM, a PhD candidate at the hospital under study, brought clinical experience and contextual familiarity (Brannick and Coghlan, 2007), but had no prior connection to the specific wards or teams involved. AMW and CO are experienced qualitative researchers (holding positions at universities), which enhanced the methodological rigour. To minimise potential bias and enhance reflexivity, reflective notes were kept throughout the research process (Czarniawska, 2007).

Following Consolidated Criteria for Reporting Qualitative Research enhanced reporting quality (Tong et al., 2007).

### 2.8 Ethical considerations

Our study was independent part of a broader, Dutch research project – 'Met VerVe' -, on how nurses care resiliently for hospital patients. –The ethical review board of [blinded for peer review] approved the research protocol Erasmus Universiteit Rotterdam (ETH2122-0079). We adhered to the Dutch Code of Conduct for Research Integrity (2018) and the Medical Research Involving Human Subjects Act (WMO).

## 3 Results

Nursing care often encounters moments when actual practice deviates from set protocols and guidelines. This situation can create challenges that necessitate adjustments or new solutions to maintain quality and safety. We identified three key triggers for such moments: 1) when standard risk assessments do not fit nursing practice; 2) when protocols and guidelines do not align with daily practices; and 3) when nurses and other healthcare professionals hold differing values on care quality and safety. For each trigger, we describe the observed behaviour, mentioned challenges and the strategies nurses used in response. This approach facilitates insights on how strategies naturally arose from specific challenges in daily practice. Table 2 illustrates how we derived our findings from the data collected.

**Table 2:** Example of thematic analysis.

**First order codes**	**Second order codes**	**Third order codes**	**Quote**
Top-down pressure through performance monitoring	Standard risk assessments do not fit nursing practice	**Trigger**	*‘All the things I****have to check and SCORE****! Really, we’ve lost track of why we’d want to do this.’ [Ward II, multidisciplinary focus group]*
Perceived irrelevance of standardised assessment			**‘I’m not the best example because I don't follow protocol.’ This nurse thinks the pain scale does not provide meaningful information because there are always patients who assign higher or lower scores than what you could expect based on their behaviou***r. According to her, using the standard pain scale once a day at the same time is not useful ‘because a number doesn’t say it all’.’ [Ward II, observation]*
Administrative formality			*‘I’m not the best example because I don't follow protocol.’ This nurse thinks the pain scale does not provide meaningful information because there are always patients who assign higher or lower scores than what you could expect based on their behaviour.****According to her, using the standard pain scale once a day at the same time is not useful ‘because a number doesn’t say it all’.’****[Ward II, observation]*
Undermining professional autonomy and ownership			*‘***There’s no point in resisting. You won’t be heard anyway***, so now my attitude is: You know what, if you want it, you get it. Fine, I’ll do it.’ [Ward I, observation]*
Tension compliance-meaningful clinical judgement			*‘Sometimes I find it so annoying to fill in the form for the sake of filling it in.****You just do it to go along with the system, to show that you’re following protocol even though [you know] the measure won’t make any difference in the care for the patient.’****[Ward I, observation]*

### 3.1 When standard risk assessments do not fit nursing practice

Nurses use various standard screening tools – e.g. Numeric Rating Scale for pain and Braden scale for pressure ulcers – aimed at proactively identifying risks so that they can intervene to prevent or solve risks. In daily practice, nurses find it challenging to act upon these assessments because a numerical score alone often fails to provide meaningful insights into the risk. A screening tool may even misrepresent the risk. More relevant information can be obtained by observing the patient’s behaviour or asking after the patient’s circumstances. As one nurse said:

*‘Mainly I rely on what I see: how the patient feels, how he reacts in the interaction with me. Is the patient tense or relaxed? Grimacing or moaning? Is he sad? What happens when he moves? Localising the pain and finding out what kind of pain is valuable.’ She links these insights to what she knows about the patient’s personal history and care pathway, and her own professional nursing knowledge. [Ward II, observation]*

This excerpt shows that screening tool metrics sometimes give a different outcome than what the nurse observes. Therefore, nurses do not always follow the agreed upon steps written down in policies (standard operating procedures, protocols, guidelines, etc.) to prevent and/or handle risk. Instead, by deviating from the system, nurses demonstrate situated resilience. One nurse explained:

*‘I’m not the best example because I don't follow protocol.’ This nurse thinks the pain scale does not provide meaningful information because there are always patients who assign higher or lower scores than what you could expect based on their behaviour. According to her, using the standard pain scale once a day at the same time is not useful ‘because a number doesn’t say it all’.’ [Ward II, observation]*

This situation is further complicated when standard risk assessment scores are monitored on the hospital management level. For instance, when process or outcome indicators of the risk assessment are shown on ward or division dashboards to give managers insight into the care quality and safety their staff provide. We observed nurses feeling under increased pressure to do these assessments, even though the scores gave only limited insight into the actual risk for a patient and required risk management. Consequently, the nurses regarded standard assessments as a formality rather than a useful tool for early risk recognition and appropriate intervention. Nurses completed forms out of obligation, feeling that this practice jeopardized their ownership and professional autonomy. One head nurse remarked:

*‘All the things I have to check and SCORE! Really, we’ve lost track of why we’d want to do this.’ [Ward II, multidisciplinary focus group]*

Our analysis identified two strategies nurses employ to navigate this front-line dilemma. The first entails seeming to comply with the system. Despite knowing that the scores have minimal impact on front-line care, nurses do standard risk assessments anyway, entering them in the patient records. This shows hospital management (monitoring the results) that nurses are complying with the system and thus stops them from getting negative scores on ward quality indicators. Non-compliance triggers automatic management instructions to fill in the forms and provide metrics. Also, seeming to comply avoids setting off alerts in the patient record. Thus, filling in forms avoids the extra administrative burden of dismissing an unnecessary alert. Describing the role of the patient record system, one nurse said:

*‘Sometimes I find it so annoying to fill in the form for the sake of filling it in. You just do it to go along with the system, to show that you’re following protocol even though [you know] the measure won’t make any difference in the care for the patient.’ [Ward I, observation]*

By seeming to comply with the system by putting in the metrics, but simultaneously ignoring the assessment scores, nurses hide their deviations from the organisational rules and regulations. Instead, working "under the radar", they develop methods that work for them. For instance, some nurses assess pain by asking a couple of questions instead of using the pain scale: "Are you in pain?" and if so, "Do you need a painkiller?" The score they enter in the patient record is based on the patient’s answers that align with the protocol for administering painkillers. This strategy of ostensibly sticking to the system (but not really) seems to arise from the nurses not being collectively heard on their views of the relevance of certain standard risk assessments for certain patients. As a result, nurses do not resist openly and seem to comply, obediently fulfilling expectations as instructed. As one nurse put it:

*‘There’s no point in resisting. You won’t be heard anyway, so now my attitude is: You know what, if you want it, you get it. Fine, I’ll do it.’ [Ward I, observation]*

The second strategy is to adopt an investigative, attentive approach, which allows nurses to circumvent standard risk assessments to gain a holistic understanding of each patient’s situation. Nurses said that they proactively seek valuable information through attentive observation and by using their intuitive knowledge and sensitivity to changes in patients’ behaviour. Nurses focus on subtle cues like facial expressions, tone of voice, and contextual factors. Nurses know when and how to ask questions to clarify their observations. This gives them sufficient tools to monitor the patient, anticipate their needs, and intervene appropriately. Nurses explained that getting to know the patient over several days, along with patients’ medical history and treatment plan or care pathway, refined their understanding and allowed for better interpretation and appropriate action. Information gathering happens continuously in every interaction with the patient, such as administering medication, bathing, bedside conversations, wound care, or simple toilet visits. Their investigative and attentive approach not only equips nurses to monitor, anticipate, and intervene effectively, it also helps to prioritise and plan care activities. Nurses described this strategy as enhancing their ability to collaborate with other disciplines to make informed treatment decisions:

*‘Well, you have four or five patients, and I plan my care activities accordingly. However, if I don't have a good feeling about a patient, they become my priority now.’ [Ward II, multidisciplinary focus group]*

This excerpt also shows that nurses rely on intuitive knowledge in risk assessment and prioritisation, such as paying attention to their own feelings of concern.

### 3.2 When protocols and guidelines do not align with daily practices

Nurses often have to deal with standard protocols that do not always align with daily practice. This mismatch can result from changes: 1) in the patient's circumstances, needs, or values, which conflict with the rules and regulations (guidelines, protocols, policies etc.); and/or 2) process variations in other wards that impact care (continuity), such as incomplete or altered steps in the care pathway; and 3) lack of available equipment.

The next example concerns a patient in the OR for knee surgery, who was administered a different spinal anaesthetic than planned, which impacted his care pathway on the ward. A nurse explains the consequences:

*‘He was given another sort of anaesthetic, diluted bupivacaine. The upside is that it keeps patient numb for longer, giving the surgeon more time to operate. The downside is that it also takes longer to wear off. And that’s what he’s experiencing right now. They [the nurses] are still hoping he can go home by 7 o’ clock [the ward's closing time]. They plan to assess his mobility by then.’ [Ward I, observation]*

We identified two strategies that nurses use to overcome these situations. First, anticipate potential changes*.* Nurses apply their investigative approach to identify potential changes in the care process and anticipate its potential impact on care planning and organisation. For example, nurses showed that they track anaesthetic techniques and procedures in the patient record to anticipate the patient's postoperative condition and consider its implications for discharge and/or ward logistics, as the following quote shows:

*‘Look, this is what I mean’, she says, pointing to the text:* General/local anaesthesia *on the form. She knows whether the procedure is painful or not. She knows from experience that when a patient gets general or local anaesthesia, that’s a lot of analgesics, which make many patients very sick, which means they have to be admitted longer. And that has consequences for logistics and planning.’ [Ward I, observation]*

Experienced nurses are adept at navigating the patient records, identifying and interpreting key information. They create their own checkpoints to monitor. Less experienced nurses, lacking on-the-job knowledge, are challenged to find and interpret the relevant information, expertise that is rarely shared among colleagues.

The second strategy is relational negotiation. At times, standard processes or treatments do not align with the patient’s immediate needs and values, causing significant short-term discomfort. This creates a dilemma for both nurse and patient. Nurses aim to find a "golden mean" through relational negotiation, which involves balancing the patient's immediate concerns with long-term objectives of the care pathway. The following quote shows a nurse engaged in relational negotiation. The patient was undergoing a six-hour intravenous treatment that during a previous admission was stopped after only two hours by the patient, against medical advice and not compliant with the care pathway or clinical guidelines. Through relational negotiation, the nurse strived to keep the patient on the intravenous infusion, while acknowledging the immediate discomfort for the patient.

*‘Patient was watching a television series on her iPad. We [researcher and nurse] watched with her for a while which was helpful for the patient. The patient said she was in great pain and wanted to stop the intravenous infusion. The nurse gave her lots of encouragement, said she could hold on for at least half an hour longer because she was such a strong woman. Patient agreed, said she would put up with the intravenous infusion until 3 o’ clock and then go home.’ [Ward I, observation]*

The nurse knew precisely how to engage with a patient, such as by watching a television series together, which made the patient feel more comfortable to say how she really felt. The nurse encouraged the patient to continue the treatment but respected her wish to stop sooner than prescribed and let the patient finish one hour before the planned time.

Another example concerns clinicians discussing evidence-based treatment strategies in the patient’s absence and only later discussing the plan with the patient. In this case the nurse acts as relational negotiator, advocating on behalf of the patient to insert the patient’s perspective, needs and values into the discussion. Based on the nurses’ holistic understanding and knowledge of the patient, this crucial role supports situated resilience:

*Both [nurse practitioner and surgeon] agree: the foot cannot be saved. That means amputation. ‘But’, the nurse interrupts, ‘[the patient] won't agree to that’. This leads to the nurse practitioner and surgeon discussing what would be a sensible alternative. After brainstorming, they came up with two scenarios to present it to the patient.’ [Ward II, observation]*

In this fragment, the nurse knows precisely what to say, when and to whom to change the situation. With a single, well-timed sentence, the nurse alters the physicians’ strategy. This results in the formulation of two alternatives better tailored to the patient's needs, values and preferences.

### 3.3 When nurses and other healthcare professionals hold differing values on care quality and safety

Nurses cooperate with many other professionals in daily practice. When nurses can share essential knowledge, skills, and authority they play a pivotal role in ensuring care quality and safety. While most collaborations are harmonious, some are not. For instance, when nurses feel their input is ignored or the other healthcare professional adheres strictly to his own rules and regulations or professional opinion. The next quote illustrates a situation where the nurse felt that her perspective was disregarded:

*‘From his own standpoint, he's totally focused on the patient's best interests. But it's just his perspective. He doesn't see or hear other viewpoints. And then you must act like a nurse.’ [Ward I, observation]*

Nurses also described situations when doctors were unaware of organisational policies, which led to doctors attempting to follow their own approach, as the next quote illustrates:

*‘The problem is that in this group [of doctors] everyone does their own thing,’ the nurse tells me. Next, she recounts the time she called a doctor to prescribe preoperative acetaminophen. ‘Oh, is that necessary?’ asked the doctor. The nurse said she had to remind him because ‘it's listed in your protocol’.’ [Ward I, observation]*

In these situations, nurses on their own are unable to uphold quality and patient safety. Yet their perspective – together with the other professionals’ knowledge, skills, and authority – is vital to tailoring care to the patient’s unique needs.

We identified two sub-strategies nurses use to bridge these situations and maintain quality and patient safety. The first is find allies, which involves nurses bypassing doctors to find alternative ways to gain the necessary knowledge, skills, or authority for the needed decisions. This often happens when nurses feel unheard and disagree with the doctor's decisions. We saw them seeking a second opinion from a nurse practitioner or another doctor, relying on their commonsense and holistic understanding. The next quote is from a phone call from a nurse to a doctor. After the doctor repeatedly ignored her, she called a co-treating doctor for the necessary knowledge to make the right decision:

*She asks the surgical resident to listen first, because she doesn't agree with his decision. ‘No’, says the resident, ‘I said nasogastric tube, didn’t I?’ Knowing the patients’ medical history, the nurse tries once again to say that she doubts this decision and firmly asks him to let her finish her story. It's all taking too long for the surgical resident, and he hangs up. The nurse decides that she should ask for another opinion and calls the internist resident. This person understands her story. Together they come up with a suitable alternative, because the nurse certainly has a good point. The patient was not given a nasogastric tube.’ [Ward II, observation]*

Nurses also report that they avoid conflict to protect themselves when they feel unsafe, especially when doctors respond to what they say with irritation.

The second sub-strategy we identified involves using various indirect means to get things done. Examples from our data include leaving notes for doctors in the patient records requesting action (‘please order acetaminophen’), placing prominent sticky note reminders on patient beds (‘Doctor: please order home medication’), and forming alliances with more influential professionals, such as asking a nurse in the Post Anaesthesia Care Unit to make sure the anaesthesiologist orders pain medication in the patient record. While indirect means avoid direct confrontation, using them can be cumbersome and yields mixed results. Sometimes they work, but nurses might still have to call the doctor they are trying to bypass to maintain quality and patient safety or to find another solution, just to get things done:

*A nurse had put a sticky note on her patient’s bed. I (researcher) ask if her reminder has been taken care of. She checks the chart several times. Still no prescription for oxycodone. ‘I think I’ll just call the assistant physician on the preoperative screening department’, says the nurse. ‘Then there’s a chance I’ll get it arranged’.’ [Ward I, observation]*

## 4 Discussion

In our study, we explored how situated resilience unfolds in everyday nursing practice. We identified three triggers where patients, their needs, values and/or interests were at risk: 1) when standard risk assessments do not fit nursing practice, 2) when protocols and guidelines do not align with daily practices, and 3) when nurses and other healthcare professionals hold differing values on care quality and safety. Nurses used various strategies to navigate these challenges effectively, enabling them to manage situations resiliently and keep safe patient-centred care a priority despite the (unexpected) obstacles they encountered.

In response to the first trigger, nurses complied with the system to avoid reprimands, applying their own methods to gain deeper insights into patients’ needs. This aligns with De Kok et al. (2023), who found that nurses “stayed under the radar” to avoid conflict. Our study suggests that their situated resilience behaviour stems from a systemic disregard of nurses’ professional insights. While ad hoc strategies help nurses manage complexity, they can reduce transparency and reinforce unsafe norms — especially when they remain unspoken (Wachs et al., 2016). Hence, embedding multidisciplinary learning in everyday practice is essential (Haraldseid-Driftland et al., 2023). Structural, safe, reflexive spaces should allow nurses to express their reasoning and adaptations, and invite collaborative learning (Lyng et al., 2022). These spaces also enhance accountability by making decisions and trade-offs visible (Haraldseid-Driftland et al., 2023; Wiig et al., 2021).

The nurses adopted an investigative approach to understand patients beyond standard assessments. By proactively noticing details overlooked by protocols, they developed a more holistic understanding of each situation. This builds on resilience research showing how nurses conduct informal assessments to anticipate change (Iflaifel et al., 2020; Lenssen et al., 2025; Tresfon et al., 2023). Examples include observing subtle signs of restlessness before restraint (Tresfon et al., 2023), or factoring in timing, medication familiarity, and patient complexity in safe medication administration (Schutijser et al., 2019; van Stralen et al., 2024). Our study adds that nurses incorporate intuitive knowledge into their assessments, prioritization, and actions — aligned with Douw et al.'s (2016) work on nurses' worry as early warning (Douw et al., 2016).

We also found that nurses contextualize formal risk scores. They do this by proactively integrating their observations and attuning to each patient’s unique situation. Risk information cannot simply be transferred between contexts in the same way; it requires adaptation to fit new circumstances. Through these adjustments, nurses show situated resilience shaped by patient-specific insights and their own sense of concern.

As for the second trigger, previous studies show that daily practice often diverges from protocols (Raben et al., 2018; Sanford et al., 2022; Vos et al., 2020), sometimes even in fundamental ways (Tresfon et al., 2023). Nurses adapt their work to ensure safety and quality despite unsupportive structures (de Kok et al., 2023; Hollnagel, 2014). Factors like time pressure, limited resources, and miscommunication contribute to these gaps (Sanford et al., 2022; Schutijser et al., 2019; Vos et al., 2020).

In our study, nurses navigated challenges by setting informal checkpoints tailored to the patients’ unique situation. They constantly balanced efficiency and thoroughness — in Safety II termed the ETTO-TETO trade-off (Hollnagel, 2009). While many studies portray resilience as a series of technical adjustments, our study shows it as a relational, patient-centred process. Lenssen et al. (2025) describe a similar process, involving proactive and investigative strategies that are inherently relational. By engaging closely with patients and their context, nurses can adapt care in a resilient, patient-centered way. We also found that nurses also negotiated with patients and physicians to find the golden mean, balancing immediate patient concerns with long-term outcomes to adapt treatment to individual patient needs, values, and interests. This aligns with acts of negotiation, such as nurses’ attentive management of relationships within informal networks, balancing short- and long-term outcomes (Drgac and Himmelsbach, 2023) to support resilient care. Mol (2008) argues, quality care means aligning knowledge, values, and patient interests in a complex, relational space — requiring moral sensitivity and compassion. This calls for not only risk awareness and organizational insight, but also relational and ethical competence to respond resiliently (Mol, 2008; Tronto, 1993; West, 2021). Such insights should inform the development of competence for resilient responding, anticipation, and monitoring, and be embedded in everyday learning opportunities — for example, through reflection on practices such as relational negotiation or investigative work (Haraldseid-Driftland et al., 2023).

Consistent with earlier studies, our findings emphasize the role of professional experience in developing situated resilience (Lyng et al., 2022; Wachs et al., 2016). However, this experience often remains implicit and unshared, especially between experienced and novice nurses. As a result, novices depend on trial and error or passive observation — risking normalization of misinterpretations or unsafe shortcuts over time potentially leading to practice drift and unsafe care (Rasmussen, 1997; Wachs et al., 2016). Learning across all levels offers valuable insights for healthcare boards, management, and policymakers to monitor and align policies and processes, and allocate the resources needed at systemic levels that will help nurses build resilience and ensure patient safety in daily practice (Wiig et al., 2023, 2021).

The third trigger involved conflicting views between nurses and other professionals on what constitutes safe, quality care. Although teamwork is seen as a sign of resilience (Iflaifel et al., 2020; Lyng et al., 2022; Wiig et al., 2023), our study shows that teamwork is not always harmonious or resilient. Nurses sometimes used indirect tactics to avoid confrontation and protect patient interests — seeking second opinions or allies. This underscores the importance of structured, reflexive, interprofessional spaces for open dialogue about differing values and perspectives — especially when values clash (Haraldseid-Driftland et al., 2023). Such spaces support shared understanding and help align professionals around the common goal of safe, high-quality care (Haraldseid-Driftland et al., 2023; Lyng et al., 2022).

### 4.1 Strengths and limitations

Data triangulation strengthened our study’s validity by combining multiple methods and respondent perspectives, offering rich insights into nurses’ practices. However, also some limitations need to be acknowledged. First, replacing the focus group for one of the included wards with three interviews, might have limited the input from that ward. However, the interviews provided valuable personal nuances without altering the overall findings.

Second, we did not include physicians in this study. However, the consistency of our findings across different professional and management perspectives suggests a strong basis for understanding key patterns.

Lastly, our study was conducted in one Dutch hospital which could limit the generalisability of the results. Nevertheless, while practices such as relational negotiation are context-specific, they reflect broader patterns of situated resilience that are not limited to a single setting. Therefore, our findings are likely to be transferable to similar healthcare settings.

### 4.2 Implications for research and practice

This study brings to light the often-invisible resilience work of nurses in everyday practice. A key strategy to support and enhance this work is the integration of mono- and multidisciplinary learning into daily routines. This requires managers to facilitate time and space during shifts, despite current pressures, and professionals to engage actively, fostering curiosity, openness, and constructive dialogue across disciplines.

Senior or quality-focused nurses can take the lead in facilitating these moments, supported by quality advisers who offer tools and coaching to embed learning in practice. Promoting such learning across all levels — including management and boards — ensures that policies, resources, and infrastructures better reflect the realities of nursing practice.

Our findings also reveal nurses’ ambition to find structural solutions, signalling their potential to contribute to system-level resilience. Further research should explore how nurses influence resilience beyond the clinical level and how their contributions can be recognised and amplified in organisational learning and improvement.

Furthermore, while we focused on the commonalities across participating departments and the study was conducted in one Dutch hospital setting, it is worthwhile to explore how challenges and adaptive strategies vary across different care contexts.

Lastly, including physicians’ perspectives in studying nurses’ resilient behaviour could further enrich the understanding of multidisciplinary collaboration and situated resilience, especially regarding nurses’ relational negotiation in response to safety challenges.

## 5 Conclusions

Our study reveals that situated resilience in nursing unfolds as a relational process where nurses’ adaptations are driven by patient-specific needs, values, and interests, and by intuition or commonsense, based on the nurses’ concern. Evidently, resilience is not simply a sequence of actions undertaken to align or fix processes. It requires attentiveness, moral awareness, and compassion, because nurses must balance individual care needs with systemic demands. Integrating multidisciplinary learning across all levels of resilience is essential to support and empower nurses and to ensure that policies, resources and training align with the realities of everyday nursing practice. Further research is needed to examine how nurses impact structure and system-level resilience.

## Funding

This research did not receive a specific grant from funding agencies in the public, commercial, or not-for-profit sectors.

## Declaration of generative AI and AI-assisted technologies in the writing process

During the preparation of this work the author(s) used ChatGPT and Perplexity to improve language (translate from Dutch to correct English) and to shorten the text (OpenAI, 2025; Perplexity, 2025).

## CRediT authorship contribution statement

**Mariëlle Van Mersbergen-de Bruin:** Writing – original draft, Project administration, Investigation, Formal analysis, Data curation, Conceptualization. **Catharina Van Oostveen:** Writing – review & editing, Writing – original draft, Supervision, Methodology, Formal analysis, Conceptualization. **Anne Marie Weggelaar-Jansen:** Writing – review & editing, Writing – original draft, Supervision, Methodology, Formal analysis, Conceptualization.

## Declaration of competing interest

The authors declare that they have no known competing financial interests or personal relationships that could have appeared to influence the work reported in this paper.

## Appendix Supplementary materials

Image, application 1

Image, application 2
