# Supplementary material for: Nurses’ challenges and strategies for safeguarding care quality and safety: A qualitative study on situated resilience
Source: Int J Nurs Stud Adv. 2025 Jun 11;9:100365. doi: 10.1016/j.ijnsa.2025.100365 (PMC12221606; doi:10.1016/j.ijnsa.2025.100365)
Supplement: Supplementary file 1 [file mmc1.docx]

*Supplementary file 1. Observation guide for non-participant observations, pain and pain management*

**General Observations**

- Observe openly and freely—capture everything seen, heard, felt, and said.
- Consider the physical space (room layout, artifacts, distances, ambiance of the room/ward).
- Note time and timing: moments of (pain) observation, administration of medication, repositioning, interventions and unexpected events.
- Pay attention to the broader nursing process and how pain-related actions connect with other care activities.
- Tip: To access the perspective of the nurse, apply the "think aloud" method—ask them to verbalize what they see, do, decide, and why.

**Pain-Related Observations**

- Patient-specific pain and pain management.
- Monitoring and assessment methods: what is said, seen, and done.
- Actions/interventions and outcomes/results.
- Disruptive or unexpected events and responses to them.
- Materials and tools used, and the nurse’s interaction with them (e.g., computer, patient records, instruments, comfort items).
- Protocols and local agreements: application and deviation.

**Interactions Related to Pain**

- Individuals involved and their roles at the moment of observation.
- Patient involvement and role.
- Behavior and positioning: body language, proximity, posture (e.g., sitting, standing).
- Interpersonal interactions: attentiveness, mutual understanding, helpfulness.
- Communication: spoken language, tone, volume, silences, empathy.
- Human–technology interactions (e.g., pagers, monitoring devices, medication administration systems).

**Quality and Safety in Pain Management**

- Identification of risks during pain assessment and treatment:
  - What is signalled, how, when, by whom, and using which information.
- Moments when pain care is effective (and thus safe):
  - What happens, what is said/done, who is involved, what methods/tools are used or absent.
- Moments when pain care is ineffective (and thus unsafe):
  - Same points as above.
- Quality and Safety management in general at the ward

**Learning, Reflection, and Improvement**

- How pain and pain treatment are discussed during care:
  - During visits, multidisciplinary meetings, informal conversations.
  - Who is involved, what is said, done, body language, tools, and information used.
- Discussion within the team or with others (e.g., during shift handovers or improvement meetings).

**Leadership and Team Dynamics**

- Collaboration and teamwork: who is involved, how does it take place, and what is done.
- Assertiveness and speaking up.
- Application of EBP, clinical reasoning, innovation, quality improvement actions, and identified “things not to do anymore.”
